# Garcinol loaded vitamin E TPGS emulsified PLGA nanoparticles: preparation, physicochemical characterization, *in vitro* and *in vivo* studies

**DOI:** 10.1038/s41598-017-00696-6

**Published:** 2017-04-03

**Authors:** Raghuvir H. Gaonkar, Soumya Ganguly, Saikat Dewanjee, Samarendu Sinha, Amit Gupta, Shantanu Ganguly, Dipankar Chattopadhyay, Mita Chatterjee Debnath

**Affiliations:** 1 Infectious Diseases and Immunology Division, CSIR-Indian Institute of Chemical Biology, Kolkata, India; 2 Advanced Pharmacognosy Research Laboratory, Department of Pharmaceutical Technology, Jadavpur University, Kolkata, India; 3 Regional Radiation Medicine Center, Thakurpukur Cancer Center and Welfare Home Campus, Kolkata, India; 4 Department of Polymer Science & Technology, University College of Science & Technology, University of Calcutta, Kolkata, India

## Abstract

Garcinol (GAR) is a naturally occurring polyisoprenylated phenolic compound. It has been recently investigated for its biological activities such as antioxidant, anti-inflammatory, anti ulcer, and antiproliferative effect on a wide range of human cancer cell lines. Though the outcomes are very promising, its extreme insolubility in water remains the main obstacle for its clinical application. Herein we report the formulation of GAR entrapped PLGA nanoparticles by nanoprecipitation method using vitamin E TPGS as an emulsifier. The nanoparticles were characterized for size, surface morphology, surface charge, encapsulation efficiency and *in vitro* drug release kinetics. The MTT assay depicted a high amount of cytotoxicity of GAR-NPs in B16F10, HepG2 and KB cells. A considerable amount of cell apoptosis was observed in B16f10 and KB cell lines. *In vivo* cellular uptake of fluorescent NPs on B16F10 cells was also investigated. Finally the GAR loaded NPs were radiolabeled with technetium-99m with >95% labeling efficiency and administered to B16F10 melanoma tumor bearing mice to investigate the *in vivo* deposition at the tumor site by biodistribution and scintigraphic imaging study. *In vitro* cellular uptake studies and biological evaluation confirm the efficacy of the formulation for cancer treatment.

## Introduction

In recent years a great deal of attention has been given to identify novel pharmacophores from natural resources that can be used to suppress cancers as well as reduce the risk of cancer development^1^. Garcinol (GAR) is a polyisoprenylated benzophenone derivative isolated from the fruit rind of *Garcinia indica* known as Kokum. Like turmeric, this plant is also used as a garnish in cooking and has been extensively used to treat gastric disorders and skin irritation^2^. Studies by different groups have revealed the potential antioxidant, antiinflammatory and anticancer effects of GAR. The anti oxidative property is due to its polyphenolic structure^3^. *In vivo* studies in animal models have shown its efficacy in suppressing azoxymethane induced colon cancer^4^, 4-nitroquinoline-1-oxide induced tongue cancer^5^, and nicotine-induced human breast cancer^6^. *In vitro* studies in different cancer cell lines reveal the efficacy of the compound in modulating cell signaling pathways involved in apoptosis and cancer development^7^. Though a large number of *in vitro* and *in vivo* studies have been done to establish its wide array of pharmacological effects, little is known about its pharmacokinetic properties and toxicity parameters. A study report claimed that oral administration of 285.71 mg/kg GAR in a mouse model bearing MDA-MB-231 did not result in systemic toxicity and mortality^7^. The compound is a hydrophobic polyphenol exhibiting extremely low aqueous solubility whereas freely soluble in common hydrophilic or organic solvents (0.344–1 g/ml). This necessitates the development of potential delivery systems to enhance its stability and bioavailability.

So far no studies have been reported regarding encapsulation of GAR in polymeric nanoparticles to improve its bioavailability and anticancer potency. The choice for an ideal polymer for nanoencapsulation is vital as it regulates the essential properties such as solubility, stability, drug loading capacity and drug release profile^8^. Poly(lactic-co-glycolic acid) or PLGA is one of the most successfully developed biodegradable and biocompatible polymer approved by Food and Drug administration (FDA) and European Medical Association for parenteral administration^9^. PLGA nanoparticles are internalized in cells through pinocytosis and endocytosis^10^.

Nanoparticle therapeutics has attracted increasing attention in recent years as an emerging treatment modality in cancer^11^ and other inflammatory disorders^12, 13^. Promising pharmacological effects of GAR have encouraged us to develop a novel garcinol nanoparticle system to improve its aqueous solubility as well as bioavailability and exploit its potential therapeutic efficacy. There are reports of using TPGS (D-α-tocopheryl polyethylene glycol 1000 succinate) as an emulsifier in nanoparticle formulation of anticancer drugs which resulted in high drug encapsulation and substantially high cellular uptake by cancer cells^14–16^.

GAR-loaded vitamin E TPGS-emulsified PLGA nanoparticles were prepared by nanoprecipitation method. The nanoparticles were characterized in terms of their particle size, polydispersity index (PDI), morphology, encapsulation efficiency (EE), zeta potential and *in vitro* drug release profile. The physical state of GAR in nanopaticles (NPs) was investigated using differential scanning calorimetry (DSC), fourier transform infra red spectroscopy and X-ray diffraction (XRD). *In vitro* efficacy assay in different tumor cell line was examined by MTT assay, apoptosis assay and DAPI staining. Cellular uptake and flow cytometric studies of FITC labeled NPs in B16F10 cell line were performed. Most importantly GAR loaded nanoparticles (GAR-NPs) were radiolabeled with technetium-99m to ascertain the biodistribution and tumor accumulation (scintigraphic imaging) in B16F10 tumor bearing mice model. The results are discussed in the following sections.

## Results

**Figure 1:**
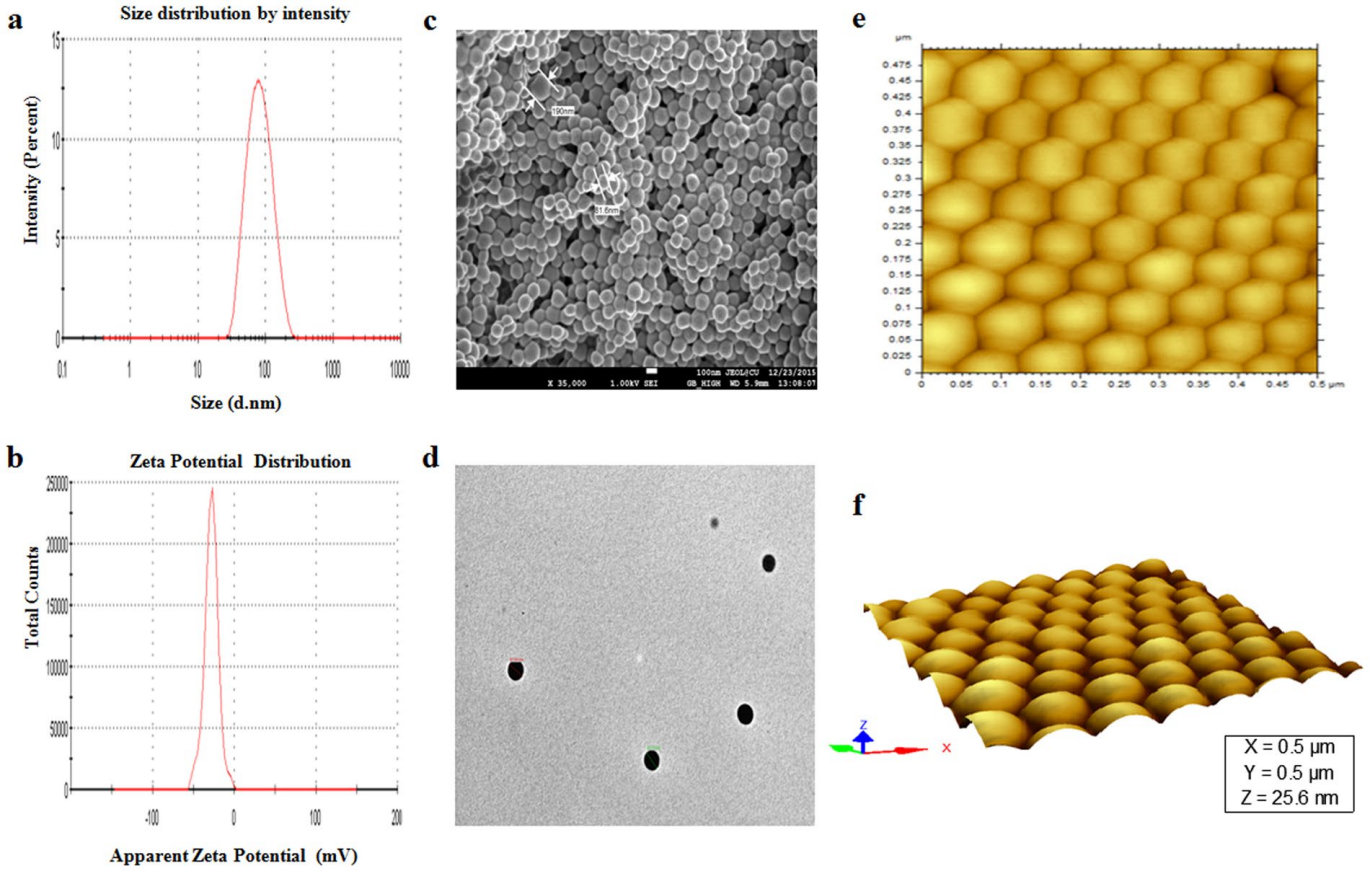
Particle size distribution, zeta potential measurement profile and microscopic imaging of GAR-NPs. (**a**) Particle size distribution pattern, (**b**) zeta potential distribution profile, (**c**) FESEM image, (**d**) TEM image, (**e**) AFM image and (**f**) three dimensional view of AFM image.

GAR encapsulated nanoparticles were developed to achieve maximum solubility and bioavailability of entrapped GAR. Blank and GAR-loaded PLGA NPs were prepared through a nanoprecipitation technique using vitamin E TPGS as an emulsifier. Table 1 summarizes average size (Fig. 1a), zeta potential (Fig. 1b), drug loading, and encapsulation efficiency of blank and GAR-NPs. Nanoprecipitation technique yielded GAR-NPs with most narrow size distribution (average diameter 88.05 ± 2.7 nm) as determined by dynamic light scattering (DLS) with a PDI value of 0.170 ± 0.05 and satisfactory zeta potential (−28.10 ± 2.1).

**Table1:** Characterizaton of GAR-NPs.

Parameters	Nanoprecipitation method			After storage at 4 °C for 3 months
Drug:Polymer ratio	1:20	1:10	1:6.66	1:10
PLGA grade	50:50	50:50	50:50	50:50
Size (nm)	90.21 ± 2.2	88.05 ± 2.7	95.45 ± 2.4	100 ± 4.2
PDI	0.212 ± 0.07	0.170 ± 0.05	0.237 ± 0.09	0.295 ± 0.08
Zeta potential (mV)	−30 ± 2.2	−28.1 ± 2.1	−33 ± 2.4	−27.2 ± 2.2
Drug loading (DL, %)	4.12 ± 1.9	9.25 ± 2.8	5.29 ± 2.1	
Encapsulation efficiency (EE, %)	53.43 ± 2.8	88.32 ± 3.3	60.61 ± 3.5

The morphology size of the GAR-NPs were determined using field emission scanning electron microscopy (FESEM), transmission electron microscopy (TEM) and atomic force microscopy (AFM). FESEM images (Fig. 1c) showed that particles were spherical with a smooth exterior. AFM studies (Fig. 1e and f) confirmed the smooth and spherical surface of the NPs along with the absence of aggregation or adhesion among NPs, three dimensional images revealed smooth spherical topography with homogeneous size distribution whereas TEM images (Fig. 1d) showed a discrete spherical outline and monodispersed size distribution of NPs. The average diameter of the NPs as obtained from FESEM (81.6 to 190 nm), TEM (87.84 to 119.13 nm) and AFM (75.46 to 125.24 nm) analyses were corroborated with the data obtained from DLS (around 88.05 nm).

The free flowing powder of GAR-NPs obtained after lyophilisation was stored at 4 °C for 3 months for particle size measurement. The particle size was slightly increased (Table 1 around 100 nm) with a moderate change in PDI value, whereas zeta potential showed the least variation; this suggested the satisfactory stability of GAR-NPs during storage.

### Determination of drug loading and encapsulation efficiency

The amount of GAR encapsulated in the nanoparticles was determined in triplicate by spectroscopic measurements. Drug loading amounts and encapsulation efficiencies of various GAR loaded nanoparticle formulations prepared by varying the amount of GAR and PLGA were measured. The highest drug loading content of GAR in GAR loaded PLGA-NPs was detected as 9.25 ± 2.8%. Encapsulation efficiency was more than 88 ± 3.3% (Table 1). GAR was encapsulated into vitamin E TPGS emulsified PLGA-NPs with a relatively high drug loading capacity and encapsulation efficiency, which might be due to the strong interaction between GAR and polymer.

### Physicochemical characterization of GAR-NPs

#### FTIR spectroscopic analysis

**Figure 2:**
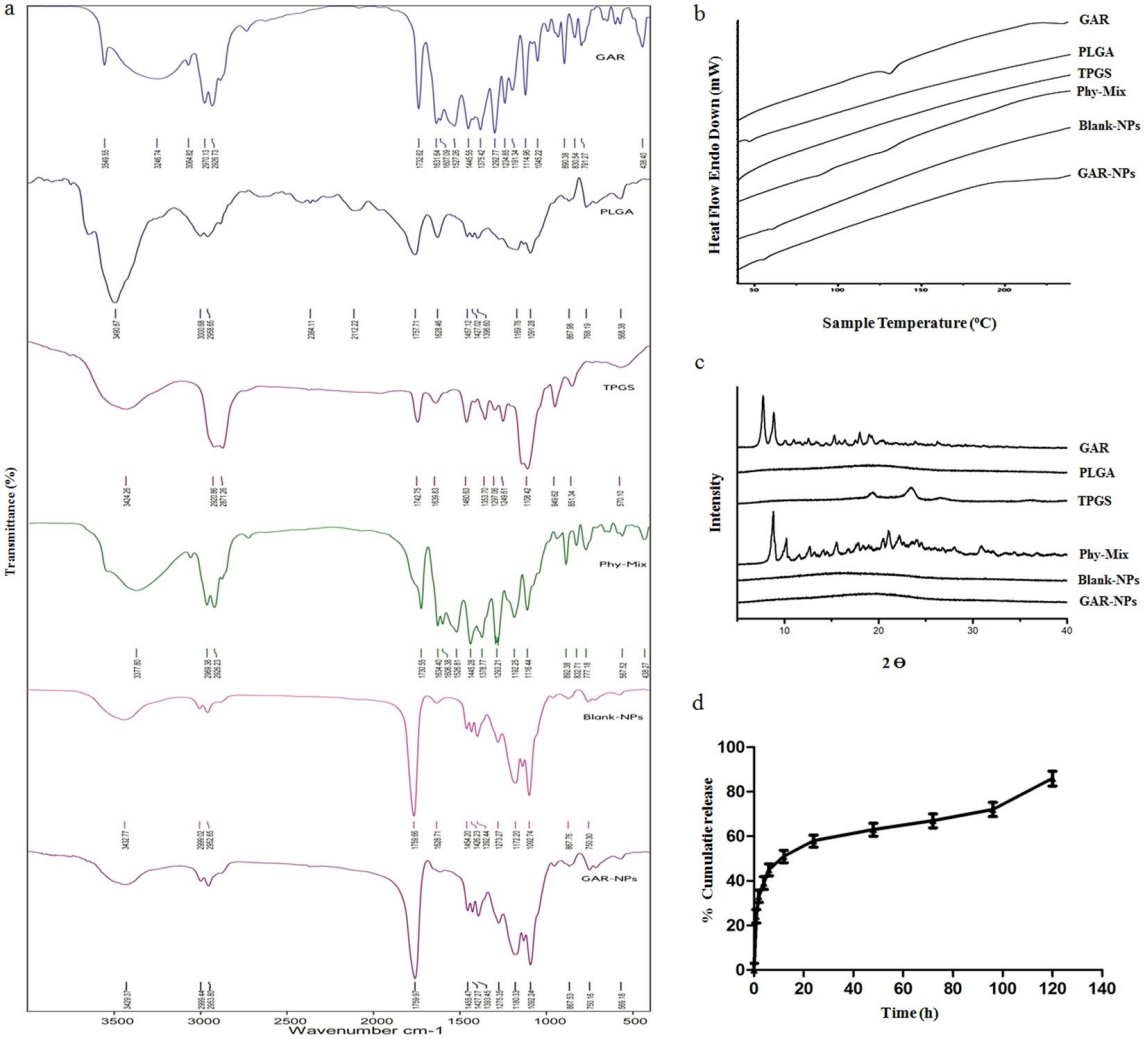
(**a**) FTIR spectra, (**b**) DSC thermograms and (**c**) XRD diffractograms of GAR, PLGA, TPGS, their phy-mix, blank-NPs and GAR-NPs. (**d**) *In vitro* drug release profile showing cumulative percent drug release of GAR-NPs vs time.

FTIR studies provide information about the stability of the GAR in the formulation, as well as the interaction between GAR and the excipients in the formulation. In this study, FTIR spectra (Fig. 2a) were recorded for GAR, PLGA, TPGS, a physical mixture of GAR, PLGA and TPGS, blank-NPs (without GAR) and GAR-NPs. As was reported earlier, the spectra revealed the characteristics peaks of GAR at 1732.62 cm^−1^ due to the presence of saturated carbonyl group, another two peaks at 1631.64 and 1607.09 cm^−1^ accounting for the presence of α,β-unsaturated carbonyl groups, and a broad band at 3246.74 cm^−1^ depicting the presence of hydroxyl function as was reported earlier^17^. The FTIR spectrum of PLGA revealed the characteristic peaks at 3490.87 cm^−1^ for hydroxyl function, 3000.68–2958.65 cm^−1^ for C-H stretching bands, and 1757.71 cm^−1^ for C-O stretching band of ester. In the FTIR spectrum of TPGS, the peak observed at 3424.26 cm^−1^ is attributed to the presence of terminal hydroxyl function while those at 2871.26 cm^−1^ and 1742.75 cm^−1^ indicate the –CH and carbonyl function stretching. Most of the characteristic peaks of GAR, PLGA and TPGS appeared in the FTIR spectrum of the physical mixture with only minor shifts. The physical interactions like hydrogen bond formation or dipole-dipole interaction existing between the functional groups of GAR and excipients resulted in the minor shifting of the peaks. The presence of the characteristic peaks of the GAR and exicipients indicates the presence of GAR in native form without any chemical interaction with the polymer and emulsifier.

#### DSC and XRD analysis

The DSC thermograms of GAR, PLGA, TPGS, their physical mixture, and GAR-NPs are shown in Fig. 2b. Free GAR and PLGA showed sharp endothermic peaks at 132 °C and 46 °C respectively. In the physical mixture the thermal behaviour of GAR and PLGA remained unchanged as evident from the DSC thermogram. This indicates that there was no reaction between the drug and the polymer. The absence of characteristic melting peak of GAR in the DSC thermogram of GAR-NPs may be due to the conversion of GAR from the crystalline state to the amorphous state or disordered crystalline state during nanoformulation which inhibited crystal growth thereby leading to enhanced stability of the formulation.

The findings from X-ray powder diffractometry study agree with the results obtained from DSC analysis. Figure 2c depicts the XRD pattern of free GAR showing distinct peaks due to its crystalline nature. The absence of peaks in the XRD pattern for PLGA confirmed its amorphous nature. The physical mixture of GAR, PLGA and TPGS exhibited a number of distinct peaks; however the positions of the peaks were somewhat different from those in GAR suggesting that minor interactions occurred between GAR molecules and the polymer matrix and emulsifier. The disappearance of distinct peaks in GAR-NPs suggested the conversion of crystalline state to amorphous state during nanoparticulate formulation.

### *In vitro* drug release study of GAR-NPs

Figure 2d depicts the drug release profile of GAR encapsulated NPs. Initial rapid release (around 39 ± 2.5%) was observed within the first 4 h. The release was moderate up to 12 h; this was followed by a slower sustained release. Around 86 ± 3.9% of GAR was released from nanoparticulated formulation over the period of 6 days. The initial burst release may be due to GAR loosely associated with the surface or embedded in the surface layer of the polymer, whereas sustained release occurred due to the diffusion, swelling/erosion and degradation of the polymeric matrix. Similar findings were also described by other workers in this field^18^.

### Cellular uptake studies

**Figure 3:**
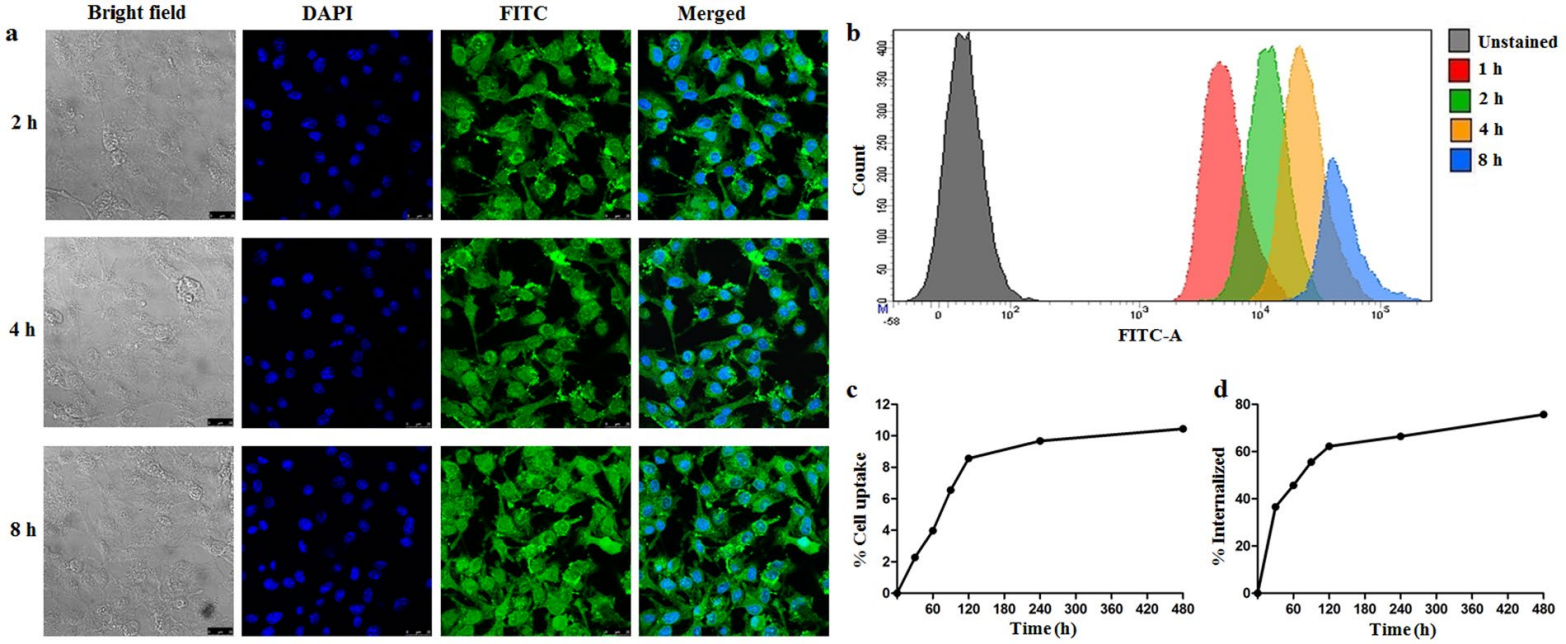
Fluorescence microscope images showing (**a**) time dependent uptake of FITC labeled NPs, cell nuclei are stained in blue while green dots represent fluorescently labeled NPs, (**b**) flow cytometric distribution of FITC labeled NPs at different time points. (**c**) *In vitro* cell binding and internalization of ^99m^Tc labeled GAR-NPs in B16F10 melanoma cells at different time intervals.

B16F10 melanoma cell line was used to confirm the intracellular uptake of fluorescence labeled NPs. FITC was used as the fluorescent dye. The result was confirmed by fluorescence microscopy observation. The cells were incubated with fluorescent-NPs for 2, 4 and 8 h, and the uptake was visualized by the presence of green signals mostly localized around the nuclei in the cytoplasm (Fig. 3a).

Cellular uptake of fluorescent-NPs was quantitated by FACS analysis, where a time dependent increase in median fluorescence intensity or MFI (Fig. 3b) was observed at different time intervals (1, 2, 4 and 8 h). Cellular uptake of the NPs is an important phenomenon. As reported by earlier workers, nanoparticle uptake by the cells is achieved through endocytosis rather than passive diffusion. It is expected that GAR entrapped in the nanoparticles can enter the cells through endocytosis. In the above studies, maximum uptake was observed at 8 h. The results clearly demonstrate the time dependent cellular uptake of NPs which will increase the intracellular concentration of GAR leading to enhanced therapeutic effect.

### *In vitro* cytotoxicity of GAR-NPs

**Figure 4:**
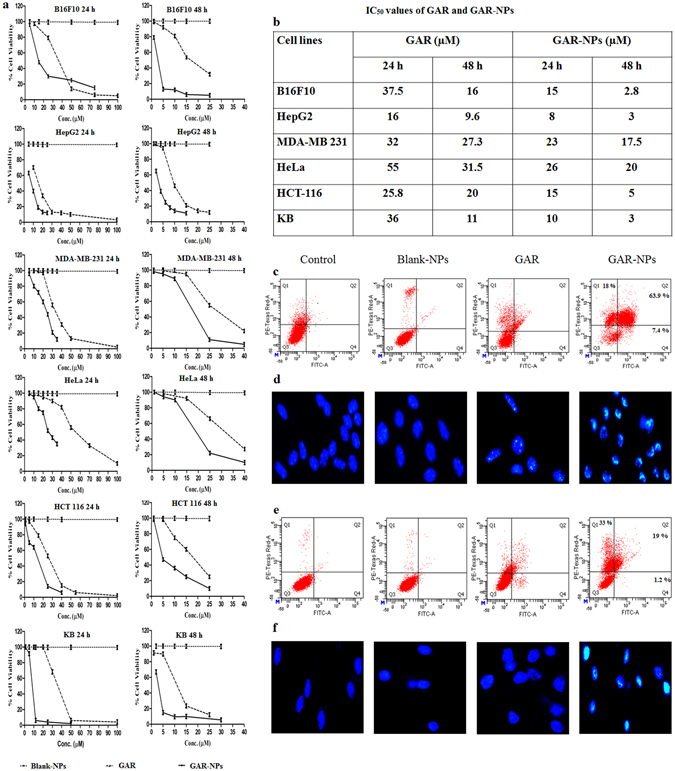
(**a**) Dose dependent cytotoxicity study results of GAR, blank-NPS and GAR-NPs against different cancer cell lines, (**b**) IC_50_ values of GAR and GAR-NPs obtained during MTT assay at 24 h and 48 h. Flow cytometer analysis of cell apoptosis using annexinV-FITC and PI staining in (**c**) B16F10 melanoma cells and (**e**) KB cells. DAPI staining showing bright fluorescence due to condensed chromatin (apoptotic condition) in (**d**) B16F10 melanoma and (**e**) KB cells.

*In vitro* cytotoxic activity of free GAR, blank-NPs and GAR-NPs was screened by MTT assay in six different cancerous cell lines (B16F10, HepG2, MDA-MB-213, HeLa, HCT-116 and KB) for 24 h and 48 h. Both free GAR and GAR-NPs exhibited concentration and time-dependent cytotoxic behaviour. Detailed analysis of cytotoxic effect (Fig. 4a) indicated that GAR-NPs elicited significantly more cell death than free GAR at an almost equivalent dose and corresponding incubation time. As depicted in the Fig. 4b, calculated IC-50 values of free GAR were much higher than those of GAR-NPs during different incubation times. Cellular internalisation of the NPs and their sustained release may be responsible for the promising cytotoxic effect. Blank-NPs did not suppress cell proliferation, indicating that the polymer matrix and emulsifier were nontoxic to tissues and cells. Various cell lines responded differently; B16F10, HepG2, HCT-116 and KB cells were found to be most sensitive to GAR-NPs. Intracellular localisation and interaction probably led to the increased cytotoxic behaviour. Anticancer potential of GAR can thus be enhanced by nanoencapsulation.

### Apoptosis assay

Apoptosis is a programmed, physiological mode of cell death which plays an important role in tissue homeostasis. In this study cell apoptosis was determined by Annexin V FITC and PI staining in B16F10 melanoma cells (Fig. 4c) and KB cells (Fig. 4e). The scatter plot has been divided into four quadrants. Q3 represents viable cells, Q4 and Q2 represent early and late apoptotic cells respectively, and Q1 indicates necrotic cells. B16F10 cells were treated with free GAR (15 µM) as well as GAR-NPs (containing 15 µM of GAR) for 24 h. The dose was 10 µM in the case of KB cells. The above mentioned doses were the IC_50_ values of GAR-NPs obtained during MTT assay. GAR-NPs induced significant apoptosis (63%, Fig. 4c) in B16F10 cells whereas relatively lower apoptosis (19%) was observed in KB cells, where the necrotic effect was more pronounced (33%). However in neither cases apoptosis induced by free GAR was significant in the above mentioned dose. Free GAR exhibited a lower effect on cellular apoptosis than GAR-NPs. Thus the nanoparticulated formulation could induce better anticancer effect than free GAR.

Apoptotic cells morphologically present the features of apoptosis following DAPI staining. The cells (B16F10 and KB) were therefore treated with blank-NPs, free GAR and GAR-NPs before DAPI staining. This revealed rounded and intact nuclei in untreated control as well as in the group exposed to blank-NPs, while the cells treated with free GAR and GAR-NPs had nuclei that appeared more brightly stained with condensed chromatin showing crescent shaped profiles around the periphery of the nucleus or separate globular structures (apoptotic bodies). The features appeared more prominent in the cells treated with GAR-NPs. It could be concluded that GAR-NPs induced apoptosis exhibiting pronounced nuclear condensation of B16F10 (Fig. 4d) and KB (Fig. 4f) cells.

### Hemolysis study

**Figure 5:**
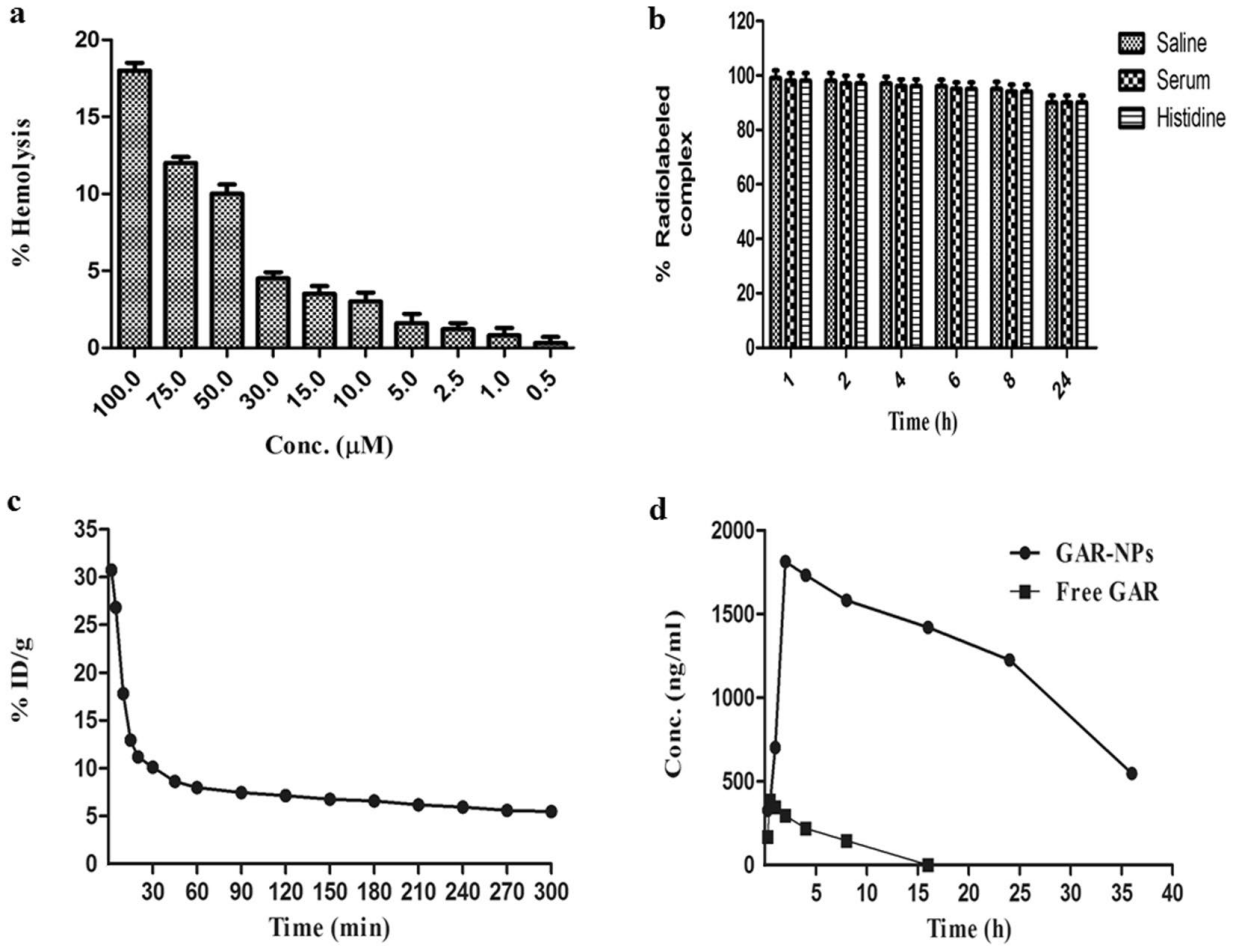
(**a**) Hemolytic activity of GAR-NPs; (**b**) stability study parameters of ^99m^Tc labelled GAR-NPs in saline, serum and histidine; (**c**) blood clearance profile of ^99m^Tc labeled GAR-NPs in rat following intravenous injection and (**d**) Comparative *in vivo* plasma concentration Vs. time profiles of GAR and GAR-NPs.

The hemato-compatibility of GAR-NPs in different concentration ranges (0.5 to 100 µM) was determined (Fig. 5a). The results exhibited negligible hemolytic activity in the formulations containing GAR between 0.5 to 2 µM, whereas significant extent (10–18%) of hemolysis (following 1 h incubation) was observed at the higher concentration range (50 to 100 µM). The study indicated negligible hemolytic activity at the tested concentration range which may be used safely for intravenous administration.

### Pharmacokinetic study

*In vivo* pharmacokinetic analysis of GAR encapsulated nanoparticles were performed in adult Sprague-Dawley (SD) rats and compared with that of the suspension of free GAR following oral administration (Fig. 5d). Different pharmacokinetic parameters including C_max_, T_max_ and AUC_0−∞_ are depicted in Supplementary Table S4. GAR-NPs exhibited sustained release of GAR over 36 h whereas aqueous suspension of free GAR exhibited rapid clearance from plasma. The presence of GAR in plasma was not detectable after 8 h following oral administration of aqueous suspension of free GAR. Oral administration of suspension containing free GAR exhibits sharp C_max_ within half an hour, whereas slow increase and sustained plasma concentration of GAR for a longer duration accompanied with significantly, delayed C_max_ occurring at 2 h was observed following oral administration of GAR-NPs suggesting an obvious sustained release of GAR from the nanoparticulated formulation. There was a marked difference in the AUC_0−∞_ between two aforementioned formulations. The AUC_0−∞_ for GAR was higher in the animals treated with GAR-NPs with a relative bioavailability of 28 as compared to an aqueous suspension of free GAR indicating improved bioavailability of GAR as a nanoparticulate suspension.

### ^99m^Tc labeling*, in vitro* cell binding and biological evaluation

In the field of nanomedicine, ^99m^Tc-radiolabeling plays an important role. ^99m^Tc can be attached to the surface of the nanoparticles and helps in understanding the *in vivo* behavior of the nanoparticulated formulation. The direct labeling approach using stannous chloride is the ideal method. Hence GAR loaded NPs were radiolabeled at neutral pH with aqueous pertechnetate using SnCl_2_-dihydrate as a reductant. All the radiolabeling parameters such as amounts of nanoparticle suspension, reductant and time of incubation were standardized to achieve the high reproducible purity that ranged between 90–92% as was verified by ITLC. ^99m^Tc labeled GAR-NPs remained sufficiently stable (≥95%) for 6 h during incubation at 37 °C with normal saline and freshly collected rat serum. A maximum of 10% of degradation occurred after 24 h of incubation showing substantially high *in vitro* stability of the nanoformulation. The level of free pertechnetate generated was about 6% following 8 h incubation with excess histidine, the value increasing to about 12% at 24 h indicating sufficient stability of the radiolabeled nanoformulation towards transchelation. From these stability studies (Fig. 5b) it could be concluded that the radiolabeled nanoformulation may exhibit enough *in vitro* stability during biological studies. The ^99m^Tc-labeled GAR-NP was also evaluated for its tumor targeting properties *in vitro* using the well characterized B16F10 melanoma cell line. Figure 3c shows cellular uptake and internalization behavior of the radiolabeled nanoformulation as a function of time. Around 2.28% of the total added activity was bound to the cells following 30 min incubation with ^99m^Tc-labeled GAR-NPs, the value becoming 10.45% after 8 h incubation. Following 30 min incubation, about 36.56% of the cell associated activity was internalized, which increased gradually to around 75.67% after 8 h. This moderately high binding and transport into the cell confirmed the findings of cellular uptake and internalization observed with unlabeled GAR-NPs during confocal microscopy studies.

Figure 5c shows the blood clearance kinetics of ^99m^Tc-labeled GAR-NPs in rat. The radiolabeled nanoformulation cleared from blood circulation in somewhat biphasic manner. The first phase exhibited comparatively rapid clearance due to biodistribution and accumulation in RES organs, whereas slow and steady clearance was observed in the second phase due to the small size of the formulation which was perhaps not readily taken up by the RES.

The evaluation of labeled and unlabeled GAR-NPs with B16F10 melanoma cells depicted the considerable amount of cellular uptake, internalization, cytotoxicity, and apoptosis. These results encouraged us to perform biodistribution studies in B16F10 melanoma tumor bearing mice to ascertain tumor localization of the nanoformulation. Table 2 summarizes the results of biodistribution studies at 2, 4, 6 and 8 h post injection of ^99m^Tc-labeled GAR-NPs. The results demonstrated rapid and efficient clearance from blood, around 0.48 ± 0.09% ID/g remaining at 8 h post injection. The accumulation of radioactivity in the liver was possibly due to the uptake of nanoformulation by RES organs. Lung accumulation was moderate. The radiolabeled nanoformulation excreted mainly through hepatobiliary route, whereas some urinary clearance was also observed.

**Table 2:** Results of biodistribution studies of ^99m^Tc labelled GAR-NPs in B16F10 tumor-bearing mice at 2, 4, 6 and 8 h post injection time periods.

Organ/Tissue	^99m^Tc Radiolabeled GAR-NPs			
	2 h	4 h	6 h	8 h
Heart	0.221 ± 0.066	0.199 ± 0.032	0.159 ± 0.066	0.145 ± 0.019
Blood*	0.710 ± 0.091	0.689 ± 0.078	0.520 ± 0.012	0.480 ± 0.091
Liver	31.310 ± 1.017	47.526 ± 0.868	48.675 ± 1.543	48.152 ± 1.261
Lung	1.563 ± 0.182	2.561 ± 0.338	3.124 ± 0.156	4.111 ± 0.165
Spleen	0.210 ± 0.039	0.351 ± 0.039	0.421 ± 0.123	0.512 ± 0.129
Kidney	2.360 ± 0.121	3.580 ± 0.589	1.960 ± 0.173	1.560 ± 0.192
Intestine	3.272 ± 0.673	7.693 ± 0.635	9.653 ± 0.820	10.918 ± 1.962
Stomach	0.185 ± 0.030	0.190 ± 0.030	0.185 ± 0.036	0.199 ± 0.046
Urine	19.193 ± 0.193	26.233 ± 1.047	22.912 ± 2.103	18.123 ± 1.126
Muscle*	0.056 ± 0.006	0.077 ± 0.002	0.076 ± 0.004	0.072 ± 0.003
Tumor*	0.221 ± 0.023	0.645 ± 0.006	0.649 ± 0.051	0.759 ± 0.063
Tumor/muscle	3.946	8.376	8.539	10.541
Tumor/blood	0.311	0.936	1.248	1.581

Results are expressed in percentage injected dose per organ/tissue (each value is the mean ± SEM of five animals per group). *Percent injected dose per gram.

**Figure 6:**
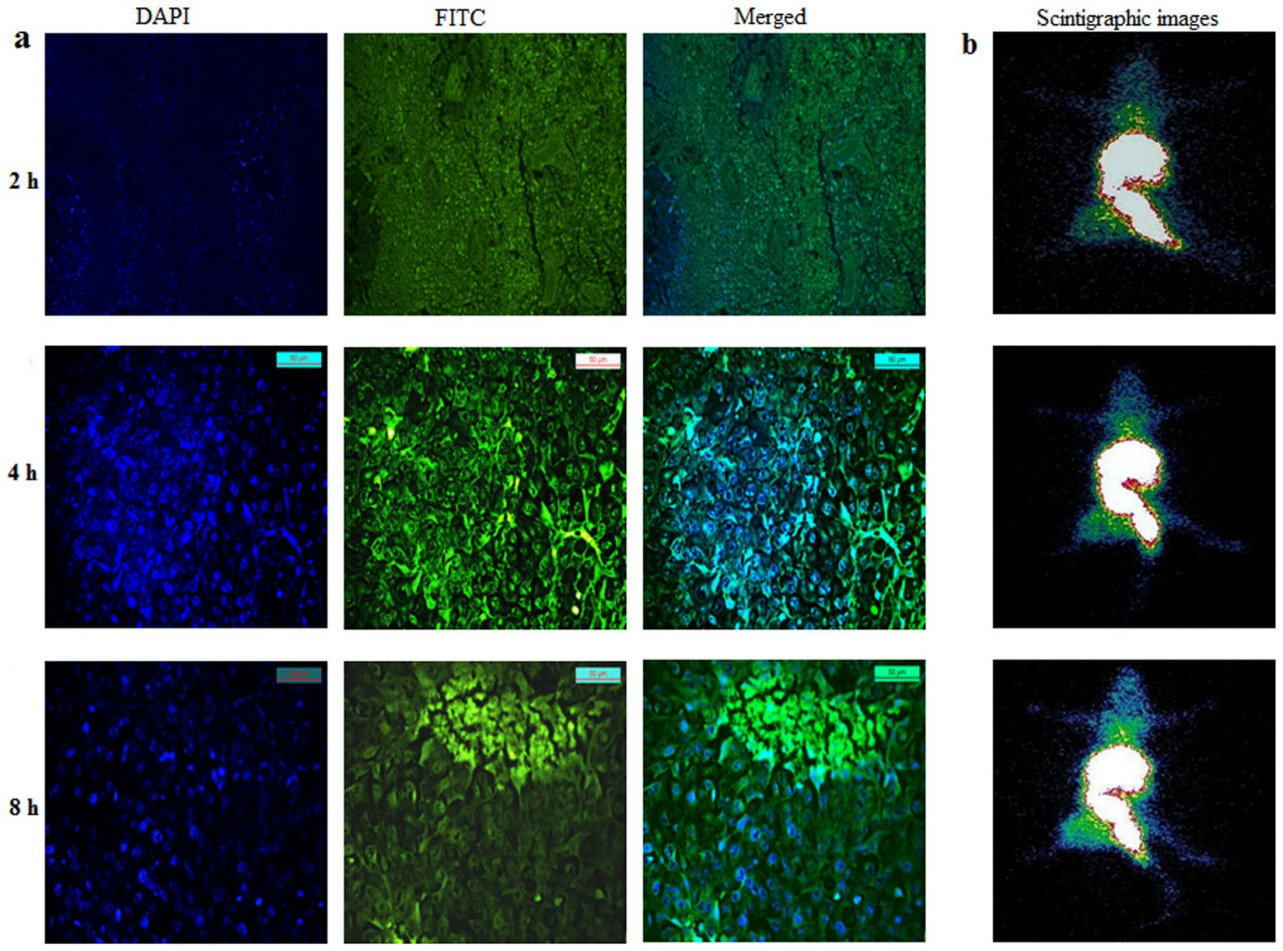
(**a**) Fluorescence images of B16F10 melanoma tumor sections excised at 2 h, 4 h and 8 h after i.v. injection of FITC labeled NP. The green color maps the distribution of FITC labeled NPs and blue color represents DAPI stained nuclei *(* **b**) scinitgraphic images of ^99m^Tc labeled GAR-NPs in B16F10 tumor bearing Balb/c mice at 2 h, 4 h and 8 h post injection time periods.

Tumor accumulation of the nanoformulation gradually increased with time. Tumor-to-muscle ratio was found to be significantly high between the studied time intervals (3.94 at 2 h becoming 10.54 at 8 h). The tumor-to-blood ratio was not very high initially (0.31 at 2 h) but improved with time (1.58 at 8 h). The uptake in other organs was also low. The radioactivity accumulation in the stomach was not very significant advocating sufficient *in vivo* stability of the labeled formulation. The rapid blood clearance and substantially high tumor-muscle ratio helped to delineate the tumor from the rest of the normal muscle during whole body tumor scintigraphy study (Fig. 6b). Intratumoral distribution of fluorescent labeled NPs (Fig. 6a) was also detected by fluorescent microscopy study indicating selective uptake of the nanoformulation in the tumor region.

## Discussion

Available literature data explains the potent cytotoxic activity of GAR and its derivatives against different human cancer cell lines. GAR exhibits emerging potential as a chemopreventive agent in carcinogenesis. However, GAR is highly hydrophobic (extremely insoluble in water) this may raise the major hurdle for the success of chemotherapy with GAR. Whereas not much effort has been put forward to solve this issue. We first report a novel GAR loaded PLGA based nanoparticulated drug delivery system using vitamin E TPGS as emulsifier. Nanoprecipitation technique produced nanoparticles of approximately 88 nm in size which is optimal for the ability of NPs to be used for intravenous administration and passive targeting to tumors. The physical characterization followed by substantially high encapsulation efficiency ensured the successful development of GAR-loaded nanoparticulated formulation. The sustained release pattern observed substantiated the use of GAR-NPs for tumor targeting. Cellular uptake study conducted using both flow cytometry and confocal microscopy, GAR-NPs showed substantially high time dependent uptake in B16F10 melanoma cell line. Time dependent cellular internalization of the nanoparticulted formulation will increase the intracellular accumulation of anticancer drugs leading to enhanced chemotherapeutic effect. The *in vitro* cytotoxicity experiments performed against different cancer cell lines demonstrated the comparatively high cytotoxic potentials of GAR-NPs than free GAR. The difference in cytotoxicity was observed for different cell line studied. IC_50_ values were considerably lower in the case of B16F10, HepG2 and KB cell lines. The results boost up the efficacy of nanoformulation in cancer treatment. The mechanism of induction of cytotoxicity in B16F10 and KB cell lines was further assured by apoptosis assay. Morphological changes of nuclei during apoptosis were observed following DAPI staining. The results revealed the inhibition of cancer cells proliferation through an apoptotic pathway. The pharmacokinetic studies revealed sustained release of GAR over a period of 36 h following oral administration of nanoparticulated suspension whereas plasma levels of GAR were not detectable beyond 8 h after administration of suspension containing free GAR. The significant increase in AUC value of the nanoparticulated formulation in comparison to free GAR suspension also indicates improved bioavailability of the nanosuspension.

Finally the tumor uptake of GAR-NPs in B16F10 melanoma tumor bearing mice was evaluated following technetium-99m radiolabeling of nanoformulation. Biodistribution and nuclear scintigraphic studies of ^99m^Tc-labeled GAR-NPs revealed moderate accumulation in tumor region. Accumulation in the tumor appeared at 2 h post injection time period and gradually increased up to 8 h. Tumor to muscle and tumor to blood ratios gradually increased with time throughout the period of study. Results obtained were further corroborated by scintigraphic imaging studies under a gamma camera. The tumor region was clearly visualized at 4 and 8 h post injection time period. The images also showed high-level of background activity in kidney, liver, GI-tract and urinary bladder (Fig. 6b). *In vivo* tumor uptake of FITC-labeled NPs were also verified by fluorescence microscopy study (Fig. 6a). This finding correlates well with the results obtained from the scintigraphic imaging study.

^99m^Tc labeled GAR-NPs also exhibited substantially high cell binding (B16F10 melanoma cells) and internalization (Fig. 3c and d). Remarkably good percentage of cell associated activity was internalized throughout the period of study. This result was also confirmed by biodistribution studies in mice bearing B16F10 melanoma tumor.

The results obtained from various *in vitro* and biological studies focused potential antitumor effect of GAR-NPs and support the use of nanosystem for GAR delivery. However this technique can be further modified to develop targeted nanoparticles which will enhance bioavailability and therapeutic efficacy through passive diffusion and active targeting to the tumor.

## Materials and Methods

### Materials

PLGA 50:50 (PURASORB PDLG 5010) was obtained as a gift from Purac Biomaterials. FITC and 4′,6-diamidino-2-phenylindole (DAPI) were purchased from Sigma-Aldrich. Vitamin E TPGS NF Grade was gifted by Antares Health Products. HPLC grade acetone was purchased from Spectrochem (Mumbai, India). All other solvents and chemicals were of analytical grade and procured from Merck, India. ^99^MoO_4_^−^ was purchased from Bhabha Atomic Research Centre (Mumbai, India) and ^99m^TcO_4_^−^ was extracted from a 5 N NaOH solution of ^99^MoO_4_^−^ by butan-2-one. ECIL gamma counter (Model LV 4755) obtained from ECIL was used for measuring tissue and organ radioactivities. Scintigraphic imaging studies of animals were done in GE Infina Gamma camera equipped with Xeleris work station.

### Extraction and isolation of GAR

The dried fruits (1 kg) of *Garcinia indica* were extracted with hexane^17^ at room temperature for 48 h. The extract was filtered and then concentrated under reduced pressure at 45 °C. The above step was repeated four times to yield the crude hexane extract (200 g), which was subjected to silica gel column chromatography (12 cm × 180 cm). Elution with petroleum ether-ethyl acetate (100:0, 95:5, 90:10, 85:15, 80:20, 75:25, 70:30, 60:40) yielded eight major fractions. Based on TLC, fraction 5 (80:20) was subjected to repetitive column chromatography over a bed of silica gel (100–200 mesh size, Merck India). Elution was done with petroleum ether-ethyl acetate (80:20) to yield pure GAR (5.5 g) as indicated by a single peak in HPLC (Supplementary Figure 1). The material was further characterized by ESI-MS, which showed a peak at m/z 625.56 attributable to M+Na^+^; (Supplementary Figure 2) the ^1^H data were also in agreement with those reported in the literature^19^ (Supplementary Figure 3).

### Preparation of GAR-loaded nanoparticles (GAR-NPs)

Nanoparticles were prepared by the nanoprecipitation method^20^. Briefly, an organic solution of PLGA (50 mg) and GAR (5 mg) in acetone (10 ml) was added to an aqueous vitamin E TPGS solution (20 ml, 0.03% w/v) under magnetic stirring at room temperature. The solvent was allowed to evaporate overnight. The suspension obtained was filtered (Whatman filter paper 1) to remove any precipitate and centrifuged at 18,000 rpm at 4 °C (Sorvall RC 5 Plus). The supernatant containing the free drug was discarded; the pellet obtained was washed 2–3 times with distilled water and lyophilised (VirTis, USA) for 48 h to get a free flowing powder.

Drug free nanoparticles were prepared according to the same procedure. Fluorescent nanoparticles were prepared by replacing GAR with FITC (5 mg).

## Characterisation of nanoparticles

### Nanoparticle yield, drug loading (DL) and encapsulation efficiency (EE)

DL and EE of the prepared nanoparticles were quantified by measuring the absorbance at 356 nm using a SHIMADZU UV-1700 UV-Vis spectrophotometer. The freeze-dried nanoparticle samples (3 mg) were dissolved in ethyl acetate (10 ml) for spectrophotometric measurement. GAR solutions of various concentrations in ethyl acetate (5–50 µg/ml) were prepared, and the absorbance at 356 nm was measured at different concentrations to generate a standard calibration curve (R^2^ = 0.998). Nanoparticle yield, drug loading and encapsulation efficiency were calculated from following three equations.

$${\rm{Nanoparticle}}\,{\rm{yield}}( \% )=\frac{{\rm{Weight}}\,{\rm{of}}\,{\rm{nanoparticles}}}{{\rm{Weight}}\,{\rm{of}}\,{\rm{polymer}}\,{\rm{and}}\,{\rm{drug}}\,{\rm{fed}}\,{\rm{initially}}}\times 100$$

$${\rm{Drug}}\,{\rm{loading}}( \% )=\frac{{\rm{Weight}}\,{\rm{of}}\,{\rm{the}}\,{\rm{drug}}\,{\rm{in}}\,{\rm{nanoparticles}}}{{\rm{Weight}}\,{\rm{of}}\,{\rm{nanoparticles}}}\times 100$$

$${\rm{Encapsulation}}\,{\rm{efficiency}}( \% )=\frac{{\rm{Weight}}\,{\rm{of}}\,{\rm{the}}\,{\rm{drug}}\,{\rm{in}}\,{\rm{nanoparticles}}}{{\rm{Weight}}\,{\rm{of}}\,{\rm{drug}}\,{\rm{fed}}\,{\rm{initially}}}\times 100$$

### Particle size distribution and zeta potential (ζ) measurements

Particle size distribution (mean diameter and polydispersity index) was determined using a Malvern Zetasizer Nano ZS (Malvern Instruments, Worcestershire, United Kingdom). Briefly, 1 mg/ml of GAR-NPs suspension was prepared in MilliQ water by sonication for 30 s. The above suspension (10 µl) was diluted to 1 ml with MilliQ water and analysis was performed. For each sample, the mean diameter of three determinations was calculated. Properly diluted samples from the above suspension were also used for the measurement of zeta potential at 25 °C using the same instrument. Values reported are the mean ± standard deviation of at least three different batches of nanoparticles.

### Field emission scanning electron microscopy (FE-SEM)

The surface morphology of GAR-NPs was observed using field emission scanning electron microscope (Jeol JSM-7600 F, Tokyo, Japan). The lyophilised samples were sputter-coated (QUORUM Q 150T ES) with platinum (4–5 nm) at a current intensity of 40 mA for 40 s. The images were captured keeping the accelerating voltage between 1–5 kV.

### Atomic force microscopy (AFM)

The shape of GAR-NPs was further characterized by AFM (5500 Agilent Technologies, Santa Clara, CA, USA). A drop (10 µl) of GAR-NPs suspension (1 mg/ml) was placed on a mica sheet. The drop was allowed to air dry for 5–10 min. The sample was further mounted on the microscope scanner. The shape was observed and imaged in ACAFM mode with frequency 166.6 kHz and scan speed 0.502 µm/s.

### Transmission electron microscopy (TEM)

The internal structure of GAR-NPs was determined by TEM (FEI, Tecnai G2 SPIRIT Bio Twin, Czech Republic). A drop (10 µl) of GAR-NPs suspension (1 mg/ml) was placed carefully on a 300 mesh carbon coated copper TEM grid. The excess solution on the grid was removed using a fine piece of filter paper and the samples were air-dried for 10 h. The dried sample was then examined at 120 kV under a microscope.

### Fourier transform infrared (FTIR) spectroscopy

In the FTIR spectroscopic study, GAR, PLGA, TPGS, the physical mixture of GAR with the excipients, and the nanoparticulated formulation with or without drug were scanned over a wavenumber range of 4000–400 cm^−1^ in an inert atmosphere in an FTIR spectrophotometer (Bruker, FTIR Tensor- 27) to understand the possible chemical interactions occurring between the drug and the polymer matrix.

### Differential scanning calorimetric (DSC) study

The physicochemical compatibility between GAR and the polymers was evaluated using a differential scanning calorimeter (Pyris Diamond TG/DTA, Perkin Elmer, Singapore). Accurately weighed samples (10 mg) of GAR, PLGA, TPGS, phy-mix, blank-NPs and GAR-NPs were sealed separately in a standard aluminium pan heated over a temperature range from 0 to 400 °C at a constantly increasing rate of 12 °C/min in an atmosphere of nitrogen gas at a flow rate of 150 ml/min.

### X-ray diffraction study

X-ray powder diffraction patterns of pure GAR, polymer and GAR-NPs were measured in an X-ray powder diffractometer (XRD-2000, Rigaku, Tokyo, Japan). The measurements were performed in the 2–50° 2θ range using CuKα radiation (45 kV, 40 mA) as the X-ray source and the rate of scanning was 1° min^−1^.

### *In vitro* release kinetics

To determine the *in vitro* release of GAR at various time intervals, accurately weighed samples of GAR-NPs (50 mg) were suspended in 5 ml of phosphate buffered saline (pH 7.4) and stirred on a magnetic stirrer (100 rpm) at 37 °C. At preselected time intervals, 1 ml of the release media was taken out and centrifuged (Spinwin MC-02, Tarson, INDIA); the supernatant was collected for analysis. To the pellet containing nanoparticles with unreleased GAR, an equal volume of fresh release media was added. The mixture was transferred to the original lot and the process continued. The collected supernatants containing released GAR were then analysed by UV-vis spectroscopy to determine the percentage of released GAR from the nanoparticles. The process was repeated thrice for each time point and the mean value was calculated. The drug release data were plotted as cumulative percentage drug release against time.

### Long term stability study

For assessing the long term stability of GAR-NPs prepared by nanoprecipitation method, these were stored at RT, 4 ± 2 °C and −20 °C for three months as per ICH guidelines. After these intervals, samples were analysed to determine particle size and zeta potential value as per the previously described method. Experiments were performed in triplicates.

### *In vitro* cellular uptake

The cellular uptake of NPs was evaluated using flow cytometry and confocal laser scanning microscopy as per reported methods^21, 22^. For flow cytometry experiments, B16F10 cells were cultured (DMEM medium) in 35 mm dish. After overnight incubation, the culture medium was replaced with fresh medium, FITC loaded NPs (50 µg/ml) were added, and incubated for 2 h, 4 h or 8 h. After incubation for specified time periods, the cells were washed thrice with cold PBS to remove unbound NPs if present, trypsinised, and centrifuged at 1500 rpm for 2 min at 4 °C. Finally cells were resuspended in 500 µl of PBS and analysed using a flow cytometer (FACS Canto II™ cell sorter, BD Biosciences) using FACS Diva software (BD Biosciences). The increase of fluorescence in the cells treated with NPs relative to that in the untreated control cells was expressed as mean fluorescence intensity relative to control.

The cellular internalization of FITC loaded nanoparticles was measured by confocal laser scanning microscopy. B16F10 cells (3.5 × 10^5^) were seeded (DMEM medium) on a cover slip placed inside a 35 mm dish and incubated for 24 h at 37 °C in humidified air containing 5% CO_2_. Following 80% confluency in each dish, the medium was removed and replaced with FITC-loaded NPs (200 µg/ml) and incubated for 2 h, 4 h or 8 h. After incubation the medium was removed and 1 ml of 70% chilled ethanol was added to each of the dishes which were kept at −20 °C for 15 min to fix the cells. The ethanolic solution was removed, washed three times with PBS, and the nuclei stained with DAPI solution for 5 min. The cells were viewed and imaged under a confocal laser scanning microscope (Leica DFC420C, Germany). The images were processed using Leica application suite software.

### *In vitro* cytotoxicity of GAR-NPs to different cancer cell lines

The cytotoxic effects of free GAR and GAR-NPs to B16F10, Hela, MDA-MB-231, HCT-116 and HepG2 cell lines were analysed by the MTT assay^23^. Briefly, B16F10, Hela, MDA-MB-231 and HCT-116 cell lines were maintained in DMEM (high glucose), whereas HepG2 was maintained in MEM medium. All cells were plated at a density of 1 × 10^4^ cells per well in 96-well plates and cultivated at 37 °C in humidified air containing 5% CO_2_ (HealForce HF-90, JAPAN). After 24 h, the respective media were removed and replaced with 200 µl of fresh media containing different concentrations of free GAR (5–100 µM for 24 h and 1–55 µM for 48 h) dissolved in minimum amount of DMSO and GAR-NPs (1–55 µM of GAR for 24 h and 1–40 µM of GAR for 48 h). After specified incubation period, media containing free GAR and GAR-NPs were removed, 200 µl of MTT solution (1 mg/ml) added to each well, and incubation carried out for another 4 h at 37 °C in darkness. After withdrawing the culture medium, 150 µl of DMSO was added to each well to dissolve the intracellular formazan crystals and absorbance was measured at 550 nm using a microplate reader (GENios, USA). Cell viability was determined as per the equation given below, where Abs_test cells_ represents the amount of formazan determined for cells treated either with free GAR or GAR-NPs and Abs_control cells_ stands for the amount of intracellular formazan present in non-treated cells.

$${\rm{Cell}}\,{\rm{viability}}( \% )=({{\rm{Abs}}}_{{\rm{test}}{\rm{cells}}}/{{\rm{Abs}}}_{{\rm{control}}{\rm{cells}}})\times {\rm{100}}$$

### Cell Apoptosis

B16F10 melanoma cells (1 × 10^6^) were cultured in 60 mm tissue culture dishes to reach 80% confluency. The cells were treated separately with different concentrations (10, 12 and 15 µM) of free GAR and GAR-NPs (containing an equivalent amount of GAR as mentioned) and allowed to incubate for 24 h at 37 °C, 5% CO_2_. Then the cells were washed with PBS and trypsinised. The detached cells were collected by centrifugation at 1000 × g, resuspended in 0.5 ml 1X cold binding buffer, treated with annexin V-FITC (1.25 µl), and incubated for 15 min at room temperature in the dark. The cells were then centrifuged (1000 × g) at room temperature for 5 min. The supernatant was removed and the cell pellet was resuspended in 0.5 ml cold 1X binding buffer. A solution (10 µl) of propidium iodide was added to the cell suspensions. Apoptotic/necrotic and living cells were detected by a flow cytometer (BD FACS) and data were analysed using FACSDiva Version 6.2 software.

### DAPI staining

This study was done to understand the apoptotic effect of blank NPs, free GAR and GAR-NPs on cell morphology^24^. B16F10 and KB cells were grown separately on cover slips placed in 35 mm dish for 24 h at 37 °C in humidified air containing 5% CO_2_. The cells were treated with blank NPs (100 µM), free GAR (15 µM) and GAR-NPs (containing 15 µM GAR) for another 24 h under the same condition as mentioned above. Then they were washed twice with PBS and fixed in 70% ethanol at −20 °C for 15 min. After washing thrice with PBS, cells were treated with DAPI solution (4 µg/ml) for 5 min at room temperature and imaged under confocal laser scanning microscope (Leica, DFC 420C, Germany). The images were processed using Leica application suite software.

### Hemolysis study

Freshly collected blood samples from male Sprague-Dawley rats were taken in heparinised tubes and centrifuged (5 min at 1000 g) at 4 °C. The supernatant was discarded and erythrocytes were washed three times with PBS (pH 7.4). The suspension (2%) thus obtained was used for hemolysis study. In order to examine the hemolytic effect, 190 µl of the suspension was added to each well of a 96-well plate and treated with 10 µl of GAR-NPs (containing an increasing concentration of GAR, e.g. 0.5, 1, 2.5, 10, 15, 30, 50, 75, 100 µM). The negative control was prepared by adding 10 µl of PBS to 190 µl of erythrocyte suspension, whereas 10 µl of TritonX-100 (10%) was added to the erythrocyte suspension in the positive control. Following incubation at 37 °C for 1 h with gentle stirring, the unlysed erythrocytes were separated by centrifugation at 10,000 *g* for 5 min and the optical density (OD) of the supernatant was measured at 570 nm. The value was compared with the OD of the supernatant (positive control) where full lysis occurred and the % lysis was calculated. The experiments were performed in triplicate to obtain the average value.

### Pharmacokinetic study

*In vivo* pharmacokinetic studies of GAR-NPs and aqueous suspension containing free GAR were done in male Sprague-Dawley (SD) rats (weight ~250 g). The animals were divided into two groups (n = 5). Animals in group-1 and group-2 were administered with aqueous suspension of free GAR and GAR-NPs respectively at a dose of 25 mg GAR/Kg body weight by oral gavage. The blood samples were collected from the retro-orbital plexus at predetermined time points (0.25, 0.5, 1, 2, 4, 8, 24 and 36 h post administration) in heparinized tubes, centrifuged (10,000 rpm for 10 mins at 4 °C) to separate plasma. To each plasma sample (0.1 ml) methanol (1 ml) was added and vortexed, the clear supernatant collected after centrifugation was filtered was filtered by passing through 0.22 µm syringe filter. Finally the samples were analysed by HPLC using X-bridge T^m^ C_18_ column (4.6 × 250 mm, 5 µm particle size) eluted with acetonitrile:water (80:20 v/v) at a flow rate of 1.0 ml/min. Detection of the drug content was carried out with waters 2489 UV/Visible detector. All the pharmacokinetic parameters were determined using Kinetica 5.1 software (Thermo Fischer Scientific). The area under the curve (AUC) was calculated using the Trapezoidal method.

### ^99m^Tc radiolabeling of GAR-NPs

GAR-NPs were radiolabeled with ^99m^Tc (technetium-99m) by direct labeling method using stannous chloride dihydrate as a reductant. Briefly, 200 µl of aqueous sodium pertechnetate (^99m^TcO_4_^−^; 185–300 MBq/ml) was added to 200 µl of GAR-NPs suspension (equivalent to 0.224 mg GAR) followed by the addition of 25 µl of freshly prepared stannous chloride dihydrate (20 mg in 10 ml nitrogen purged water containing 100 µl of 6 N HCl) and incubation for 15 min at room temperature. The radiolabeled solution was stored at room temperature in shielded vials for subsequent studies. The radiolabeling efficiency was determined by ascending instant thin layer chromatography (ITLC) using 2.5 × 10 cm silica gel coated aluminium sheet as the stationary phase and acetone as a mobile phase as per the previously reported procedure^25^.

### *In vitro* stability studies of radiolabeled GAR-NPs

The stability of ^99m^Tc-labeled nanoformulation was ascertained *in vitro* as per the previously reported method^25^. Briefly, the radiolabeled nanoformulation (0.1 ml) was added separately to 0.9 ml of (a) normal saline, (b) serum, and (c) histidine solution (10^−2^ M). The resulting mixtures were incubated at 37 °C for 24 h. Samples were withdrawn from the mixture at various time intervals (0, 1, 2, 4, 6, 8 and 24 h) and analysed by ITLC (described above) to check for any dissociation of the radiolabeled formulation.

### *In vitro* cell-binding and internalization of ^99m^Tc-labeled GAR-NPs

The cell-binding and internalization of ^99m^Tc-labeled GAR-NPs into B16F10 mouse melanoma cell lines was studied according to the method described previously with some modifications^26^. Briefly, 1 × 10^6^ cells were cultured in a 35 mm dish with DMEM medium at 37 °C for 24 h. After the addition of about 37 kBq of ^99m^Tc-labeled GAR-NPs to the medium, the cells were incubated at 37 °C in 5% CO_2_ for different time intervals (0.5, 1, 2, 4, 6 and 8 h). After desired time interval the medium was removed to stop cellular internalization and cells were washed with ice-cold phosphate-buffered saline (pH 7.2). The radioactivities in cell pellet (total bound) and the supernatant (unbound) were measured in a gamma counter (Electronic Corporation of India, Model LV4755, Hyderabad). Cell surface-bound radioligand was removed by washing the cells with ice cold acidic buffer (0.05 mol/l glycine solution, pH adjusted to 2.8 with 1N HCl) for 5 min at 0 °C. The cell-associated radioactivity that was not removed by this procedure was taken as the internalized radioactivity. Finally, cells were treated with 1N NaOH at 37 °C for 10 min to detach them from plates and centrifuged. The supernatant was collected and the radioactivity was measured. The percent of internalized radioactivity was calculated according to the method described previously. All experiments were repeated thrice and the results were expressed as the mean ± standard deviation.

## ***In vivo*** evaluation of ^99m^Tc-labeled GAR-NPs

### Blood clearance study in rats

Blood clearance of ^99m^Tc-labeled GAR-NPs was studied in healthy male Sprague-Dawley rats weighing around 250–300 g (n = 3). To anesthetized and well-hydrated animals, ^99m^Tc-labeled nanoformulation was injected through one femoral vein. At preset time intervals (ranging from 2 min to 8 h) blood samples (0.5 ml) were collected from the other femoral vein, weighed accurately and analysed in a gamma counter to measure the radioactivity in the sample. The percentage of injected dose/g of the blood sample (% ID/g) was calculated and plotted against respective time interval to generate the blood disappearance curve.

### Biodistribution in mice

*In vivo* biodistribution studies were performed in B16F10 tumor bearing Balb/c mice (body wt 25 ± 1.5 g) as per the previously reported method^25^. Freshly collected B16F10 cells (2 × 10^7^ cells) suspended in saline were injected intramuscularly to the thigh of the right hind leg of each mouse. After two weeks a palpable tumor (volume 1 ± 0.1 cm^3^) developed and the animals were used for biodistribution studies. To each of the well hydrated animals ^99m^Tc-labeled GAR-NPs (0.03 ml, 8–12 MBq/kg) were injected through the tail vein. At 2, 4, 8 and 24 h post injection, the animals (n = 4) were euthanized and blood sample was collected by cardiac puncture. Urine along with urinary bladder as well as other organs was also collected. All the samples were collected in pre-weighed scintillation vials and radioactive counts were measured in a gamma counter. Organ uptake was expressed as a percentage of the injected dose per gram of tissue (% ID/g) or per organ.

All animal care and experiments were carried out as per guidelines laid down by the CSIR-IICB Animal Ethics Committee (IICB-AEC; Registration Number: 147/1999/CPCSEA) registered under Committee for the Purpose of Control and Supervision of Experiments on Animals (CPCSEA). Government of India, New Delhi. All animal studies and experimental protocols were approved by IICB-AEC (Ref.: IICB/AEC-APP/August-Meeting/2013).

In a separate experiment, B16F10 tumor bearing mice were injected with FITC labeled NPs suspended in PBS at a concentration of 40 mg/kg body wt via tail vein^27^. Animals were sacrificed at 2, 4 and 8 h post injection (p.i.) time period. Tumors were dissected out washed thrice with PBS and stored in 10% formaldehyde at room temperature for 24 h. The tumor tissue was embedded in paraffin and cut into 5-µM-thick sections, and the nuclei were stained using DAPI. The intratumoral distribution of FITC labeled nanoformulation was imaged using fluorescence microscope (Leica, DFC420C, Germany).

### Gamma scintigraphy

The tumor bearing mice were anaesthetized (intramuscular ketamine injection), ^99m^Tc-labeled nanoformulation (100 µl, 3.7 MBq) was administered through the tail vein, placed under GE Infinia Gamma Camera equipped with Xeleris Work Station, and static images were acquired at 2, 4, and 8 h post-injection.

### Statistical analysis

All experiments were repeated for at least three times, each time on a different day. All mean values of animal experiments are expressed as %ID per g of tissues or organs ± SD. Statistical analysis was performed by one-way ANOVA. Graphs were prepared using GraphPad Prism 5 and ORIGIN 8.0.

## Electronic supplementary material

Supplementary info
