# Supplementary material for: Garcinol loaded vitamin E TPGS emulsified PLGA nanoparticles: preparation, physicochemical characterization, in vitro and in vivo studies
Source: Sci Rep. 2017 Apr 3;7:530. doi: 10.1038/s41598-017-00696-6 (PMC5428804; doi:10.1038/s41598-017-00696-6)
Supplement: Supplementary file 1 — Supplementary info [file 41598_2017_696_MOESM1_ESM.pdf]

Garcinol loaded vitamin E TPGS emulsified PLGA nanoparticles: preparation, physicochemical characterization, *in vitro* and *in vivo* studies

Raghuvir H. Gaonkar[1,+], Soumya Ganguly[1,+], Saikat Dewanjee[2], Samarendu Sinha[3], Amit Gupta[3], Shantanu Ganguly[3], Dipankar Chattopadhyay[4] and Mita Chatterjee Debnath[1,]*

[1]Infectious Diseases and Immunology Division, CSIR-Indian Institute of Chemical Biology, Kolkata, India.
[2]Department of Pharmaceutical Technology, Jadavpur University, Kolkata, India.
[3]Regional Radiation Medicine Center, Thakurpukur Cancer Center and Welfare Home Campus, Kolkata, India.
[4]Department of Polymer Science & Technology, University College of Science & Technology, University of Calcutta, Kolkata

* Corresponding author, M.C.D. (mitacd@iicb.res.in)
+ These authors contributed equally to this work.

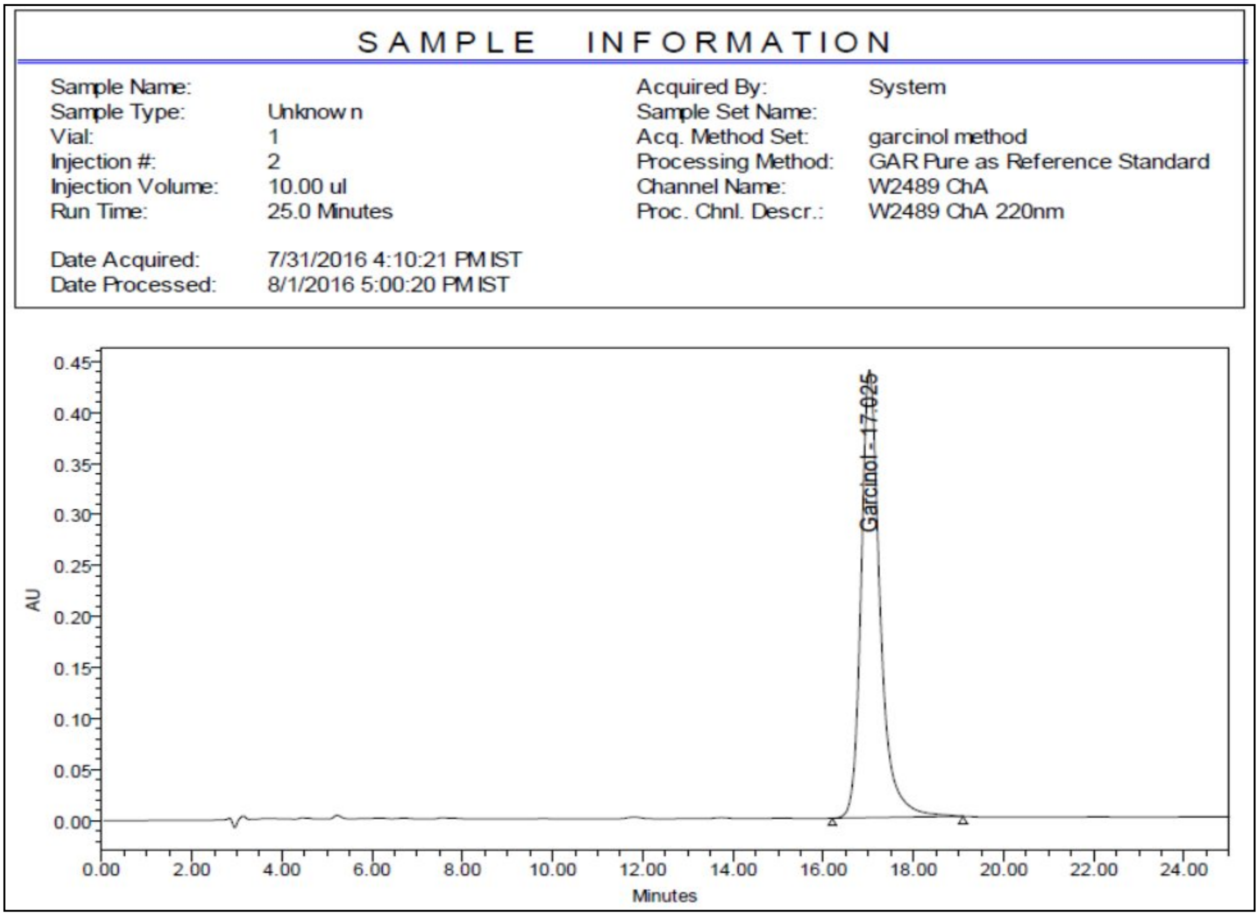

Supplementary Figure S1. HPLC chromatogram of GAR.

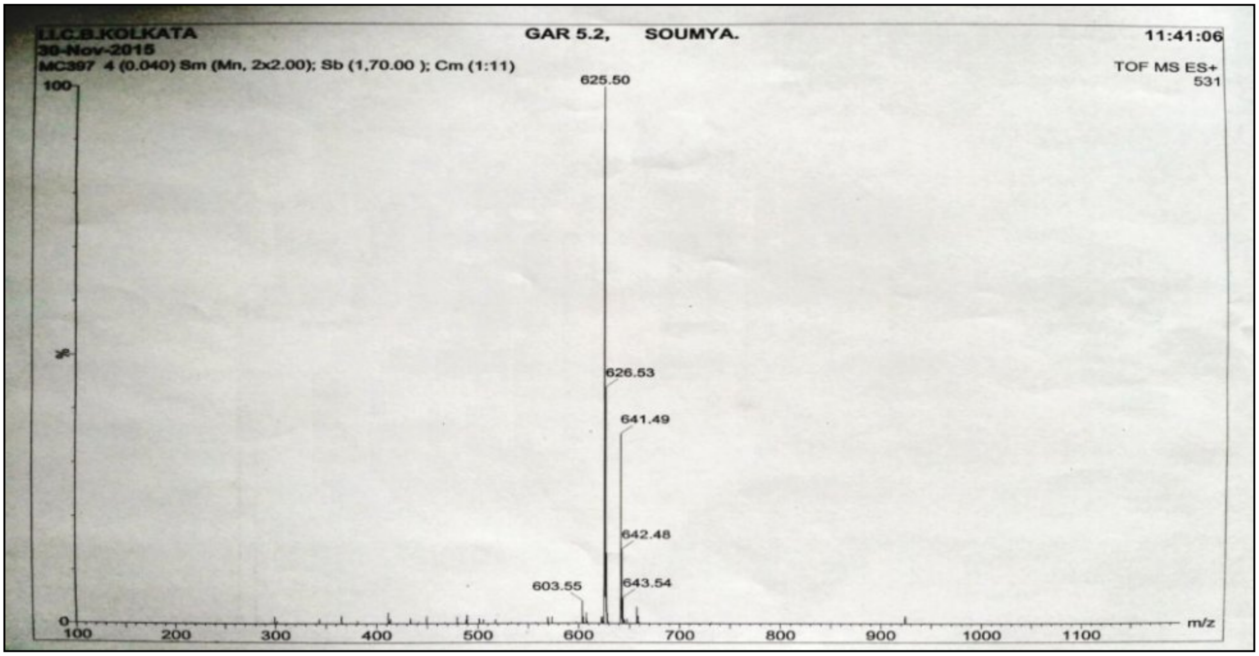

Supplementary Figure S2. ESI-Mass spectra of GAR.

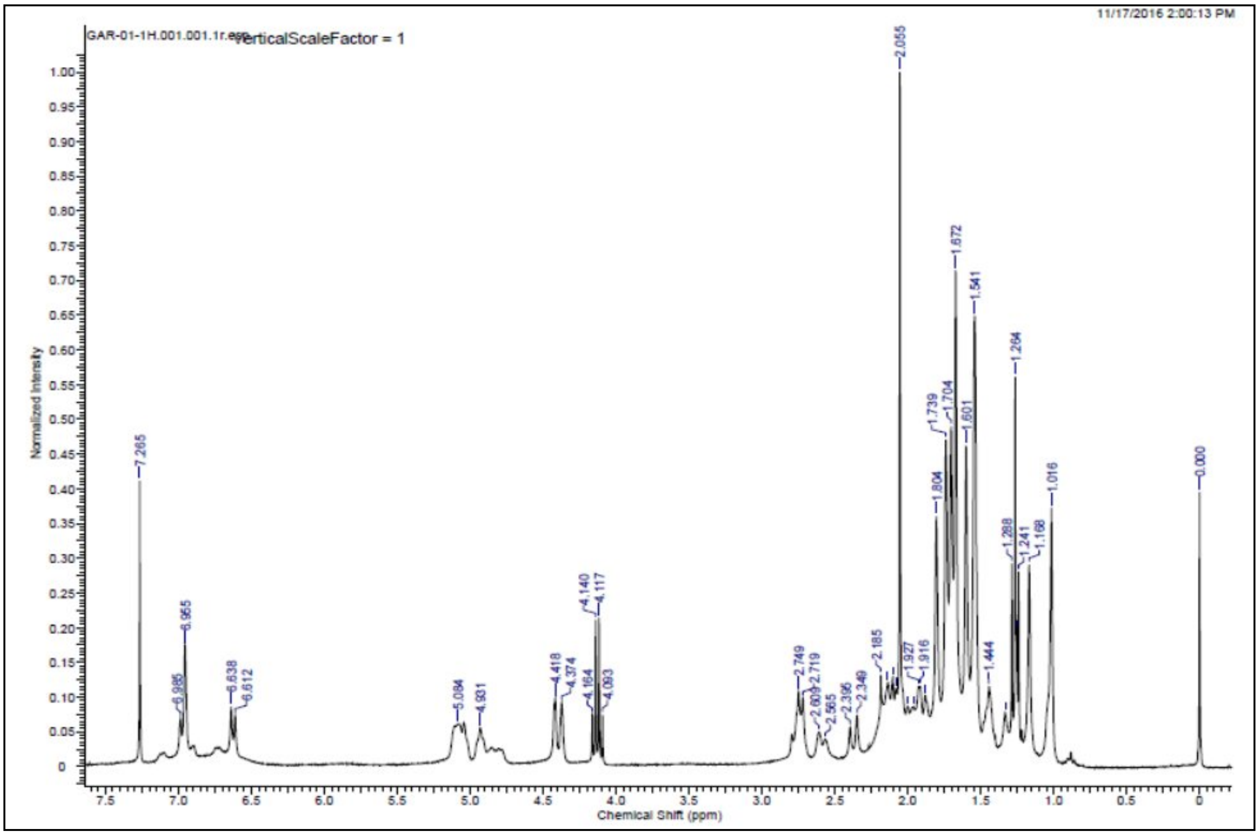

Supplementary Figure S3. ^{1}H NMR spectra of GAR.

	**GAR**	**GAR-NPs**
**Route of Administration**	Oral	Oral
**Dose (mg/kg b wt)**	25	25
**C_{max} (ng/ml)**	385	1814
**T_{max}(h)**	0.5	2
**K_{el}(h^{-1})**	0.161	0.017
**$t_{1/2}$(h)**	4.3	40.76
**$AUC_{o-\infty}$ (h.ng/ml)**	2721.45	77253.8
**F_{rel}(with respect to GAR-NPs)**	-N/A-	28.386

Note: Data for Cmax are represented as mean ± SD (n=5).
Abbreviations: C_{max}, maximum concentration; T_{max}, time at C_{max}; AUC, area under the curve; FRel, relative bioavailability; b wt, body weight.

Supplementary Table S4: Pharmacokinetics parameters of free garcinol (GAR) and garcinol-loaded nanoparticles (GAR-NPs).

**Solvents**	**Volume of Solvent required to dissolve 100 mg GAR (ml)**	**Comments**
Acetone	0.170 ± 0.32	Freely soluble
Methanol	0.140 ± 0.16	Freely Soluble
Ethanol	0.165 ± 0.23	Freely Soluble
Chloroform	0.090 ± 0.41	Very Soluble
Dichloromethane	0.290 ± 0.39	Freely Soluble
Ethyl Acetate	0.210 ± 0.28	Freely Soluble
Distilled Water	1015 ± 1.42<br>(Remains as a suspension)	Insoluble

Supplementary Table S5: Solubility studies of GAR as per USP specifications (The Studies have been done in triplicate).
